# Demand and Predictors for Post-Discharge Medical Counseling in Home Care Patients: A Prospective Cohort Study

**DOI:** 10.1371/journal.pone.0064274

**Published:** 2013-05-30

**Authors:** Shih-Tan Ding, Chuan-Lan Wang, Yu-Han Huang, Chin-Chung Shu, Yu-Tzu Tseng, Chun-Ta Huang, Nin-Chieh Hsu, Yu-Feng Lin, Hung-Bin Tsai, Ming-Chin Yang, Wen-Je Ko

**Affiliations:** 1 Division of Hospital Medicine, Department of Traumatology, National Taiwan University Hospital, Taipei, Taiwan; 2 Department of Nursing, National Taiwan University Hospital, Taipei, Taiwan; 3 Graduate Institute of Clinical Medicine, College of Medicine, National Taiwan University, Taipei, Taiwan; 4 Institute of Health Policy and Management, College of Public Health, National Taiwan University, Taipei, Taiwan; 5 Department of Surgery, National Taiwan University Hospital, Taipei, Taiwan; Johns Hopkins Bloomberg School of Public Health, United States of America

## Abstract

**Rationale:**

Post-discharge care is challenging due to the high rate of adverse events after discharge. However, details regarding post-discharge care requirements remain unclear. Post-discharge medical counseling (PDMC) by telephone service was set-up to investigate its demand and predictors.

**Methods:**

This prospective study was conducted from April 2011 to March 2012 in a tertiary referral center in northern Taiwan. Patients discharged for home care were recruited and educated via telephone hotline counseling when needed. The patient’s characteristics and call-in details were recorded, and predictors of PDMC use and worsening by red-flag sign were analyzed.

**Results:**

During the study period, 224 patients were enrolled. The PDMC was used 121 times by 65 patients in an average of 8.6 days after discharge. The red-flag sign was noted in 17 PDMC from 16 patients. Of the PDMC used, 50% (n = 60) were for symptom change and the rest were for post-discharge care problems and issues regarding other administrative services. Predictors of PDMC were underlying malignancy and lower Barthel index (BI). On the other hand, lower BI, higher adjusted Charlson co-morbidity index (CCI), and longer length of hospital stay were associated with PDMC and red-flag sign.

**Conclusions:**

Demand for PDMC may be as high as 29% in home care patients within 30 days after discharge. PDMC is needed more by patients with malignancy and lower BI. More focus should also be given to those with lower BI, higher CCI, and longer length of hospital stay, as they more frequently have red flag signs.

## Introduction

Current global ageing is continuously progressing [1], [2] and hospitalization demands increase every year [3]. For in-patient care, hospitalist medicine continues to grow and is now mainstream treatment [4]–[8]. But even though the hospitalist system reduces hospitalization costs, a major concern is the continuity of patient care, which, when interrupted after discharge, correlates with adverse events and leads to readmission [9], [10].

In fact, readmission rates are very high for elderly patients and are reported to be around 20% within one month after discharge [7], [11]. Several possible reasons include instability of chronic disease and insufficient communication among physicians [12], yet the probability of post-discharge requirements for help and their classifications are not very clear. Patients with different types of requirements deserve different types of transitional care.

Thus, post-discharge medical counseling (PDMC) using the telephone was set up to investigate the needs for medical help and provide support before the occurrence of readmission, especially for patients cared for at home. Predictors and their association with adverse events after discharge were also analyzed.

## Methods

### Study Subjects

This prospective study was conducted at the National Taiwan University Hospital, a tertiary-care referral center in northern Taiwan. From April 2011 to March 2012, all patients aged >20 years old and admitted to the hospitalist-care ward were consecutively screened. In-patient care was performed by hospitalists. In Taiwan, the hospitalist-care system and primary-care physicians system for in-patient care exist together currently [7]. Patients admitted to one-half of the ward were recruited because another investigation was conducted in the other half. Potential participants from patients who were discharged alive for home care were identified. Other eligibility criteria included having a telephone line at home without communication deficits.

Patients were excluded if they were repeatedly admitted, required subspecialty care, or were enrolled in another post-discharge study. The study was approved by the Research Ethics Committee of National Taiwan University Hospital. All participants provided written informed consent.

### Study Protocol

When up for discharge, the enrolled patients and their caregivers were educated regarding the standard care plan and post-discharge monitoring methods based on their illness, such as blood glucose measurement for diabetes mellitus. They were given the contact information of the PDMC that they could call if they needed clinical advice. The telephone hot-line for the PDMC was free and was maintained from 8 a.m. to 8 p.m. every workday and 8 a.m. to 5 p.m. on the weekend.

In the PDMC contact, the case manager who had a nursing certificate took first-line responsibility for responding to the counseling needs, which included health/diet/drug instructions, care skill explanations, and clinic appointments. If the problem was beyond the ability of the case manager, she called for help from a nurse practitioner and then an attending physician until the issue was resolved. The case managers could choose to call an on-call attending physician directly if a red-flag sign was present. Red-flag signs (RFS) (Table 1), indicated a worsening condition and were defined by a consensus in a round table meeting as outlined in a previous study [13]. These RFS consisted of 1) abnormal vital signs like high blood pressure, fever, tachycardia, and dyspnea; 2) abnormal findings related to daily care, including consciousness level, BI score, stool, sputum, urine output, and body weight; and 3) progression of specific findings such as edema, and local skin lesion. If further medical care was required, the patient was referred to the clinic or emergency department because in Taiwan, prescriptions are given only after a face to face visit.

**Table 1:** Criteria of red flag signs for each specific indicator.

Indicator	Worsening criteria
Body temperature	≥38°C
Heart rate	>120 bpm or <60 bpm
Blood pressure	Systolic pressure >180 mm Hg, diastolic >105 mm Hg
Dyspnea*	Increase in dyspnea score by >1 grade
Consciousness	decrease in Glasgow coma scale by >1 grade
Barthel score	<60% of baseline [25]
Stool	Black or bloody
Sputum	Bloody or purulent
Urine output	<60% of baseline daily amount or 0.5 ml/Kg/hr [26]
Body weight	Weight gain ≥2 Kg [27]
Blood glucose	>400 mg/dL anytime or >250 mg/dL AC
Leg edema#	Increase in edema score by >1 grade
Pain†	Increase in pain scale by >2 or pain scale >4
Size of local lesion‡	Increase in size by 20%

This table is modified from our previous study [13] with permission from publisher of BioMed Central.

* Measured by the Medical Research Council dyspnea scale [28].

# Measured by a grading system developed for cancer treatment [29].

† Measured by the Numerical Rating Scale [30].

‡ Measured by the longest length of the lesion.

### Clinical Characteristics

The patients’ clinical characteristics, laboratory data, hospital course, and outcomes were recorded by the case managers. A unified case report form containing a default option for selection was used in order to avoid ambiguous data coding. The primary endpoints were the request for PDMC and the presence of RFS. Other outcomes after discharge included unplanned visits to the emergency department and unplanned readmission.

The Charlson co-morbidity index (CCI) and Barthel index (BI) scores were calculated as in previous studies [14], [15]. A BI score >70 was defined as independent performance of activities of daily life, ≤35 as dependent, and the remainder as intermediate grade. Underlying malignancy was defined as active cancer without mention of cure or remission. Anemia was defined as hemoglobin <12 g/dL in males and <11 g/dL in females. A primary care physician was defined when the patient visited the same doctor three times or more within one year prior to this admission [9]. Artificial tube/catheter included naso-gastric tube, tracheostomy tube, draining tube, Foley catheter, and catheter for dialysis.

### Statistical Analysis

Inter-group differences were compared using independent *t* test for numerical variables and chi-square test for categorical variables. By the stepwise method, multivariate Cox proportional hazard regression was used to identify factors associated with demand for PDMC. A two-sided *p*<0.05 was considered to be significant. Survival curves were generated using the Kaplan-Meier method and compared using the log-rank test. All analyses were performed using the SPSS (Version 15.0, Chicago, IL).

## Results

**Figure 1:**
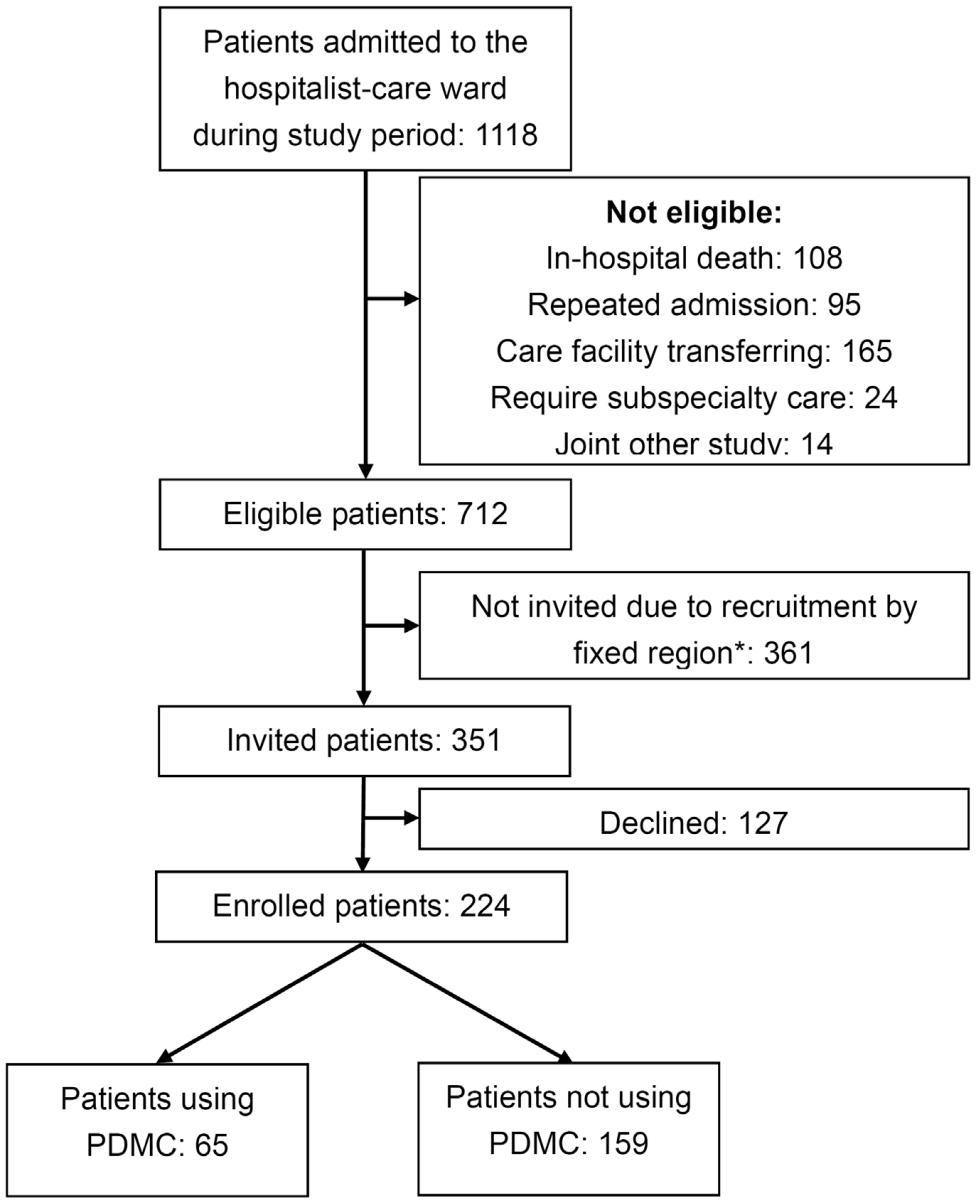
Flow chart of patient enrollment. *Patient admitted in fixed region of the hospitalist-care ward.

From April 2011 to March 2012, 1118 patients were admitted from the emergency department to the hospitalist-care ward. Of the 712 patients discharged for home care, 351 eligible patients were invited and 224 finally enrolled (Fig. 1). Within 30 days after discharge, PDMC were requested 121 times from 65 (29%) patients. Among them, RFS was found in 17 events from 16 (7%) patients.

**Figure 2:**
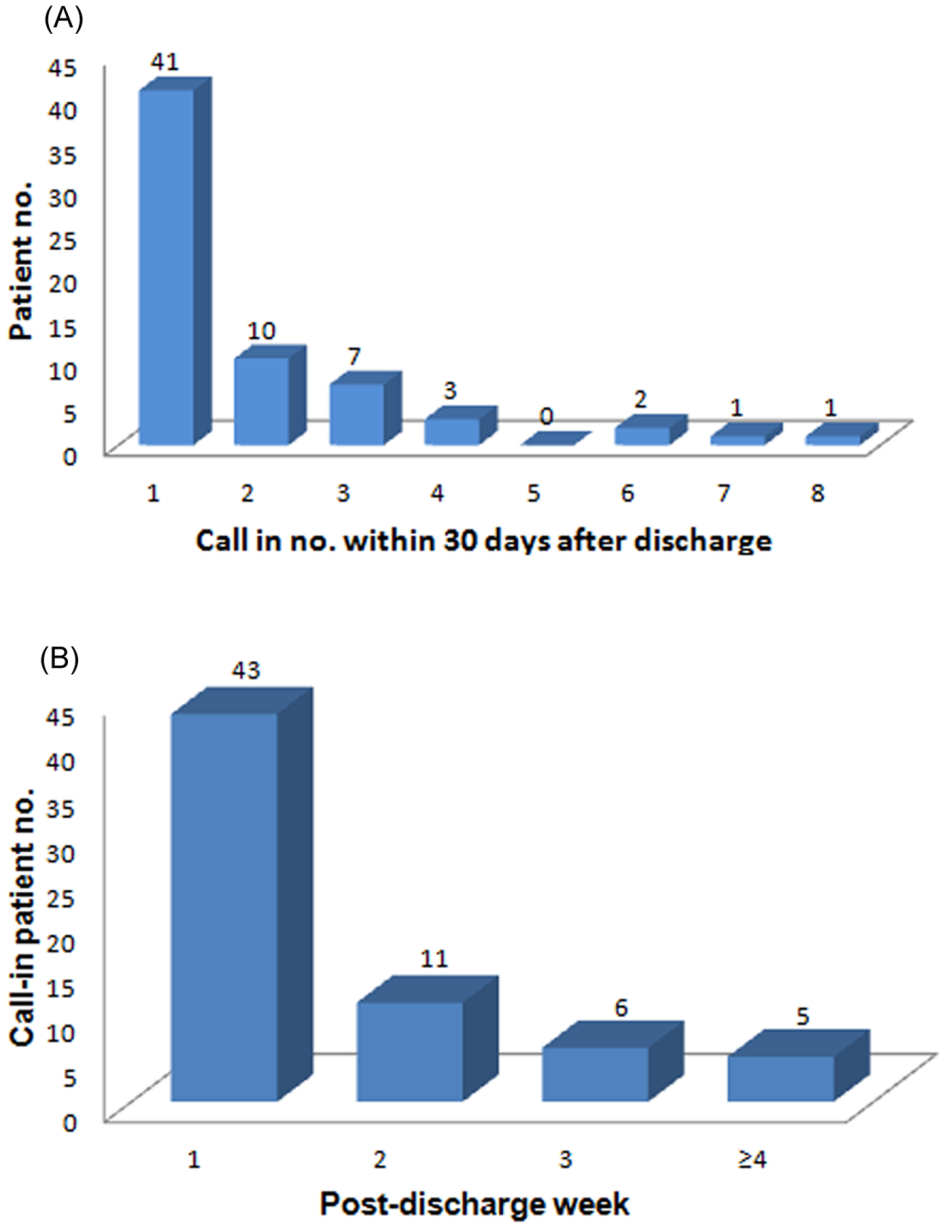
Bar charts of the (A) pattern of call-in numbers per patient and (B) timing of call-in service used.

Of the 65 patients who used PDMC, 24 (37%) called more than once and 4 (6%) called more than five times (Fig. 2A). The PDMC was needed mostly in the first week post-discharge, and then decreased thereafter (Fig. 2B). In terms of the nature of the call-in, the issues identified were problems related to major illnesses in the last admission (n = 45; 37%), new symptoms/problems (n = 15; 13%), and questions unrelated to changes in medical illness (n = 61; 50%), which include 26 for examination/clinic arrangements, 5 for certification, and 14, 11, and 5 for general, drug, and tube/wound instructions, respectively. Advice and explanations were given to all patients. Later, 31 patients (26%) were asked to receive clinical services.

There were 16 patients with 17 PDMC plus RFS, which occurred with a mean of 8.2 days post-discharge. These included 13 events associated with major illness on the last admission and 4 with new symptoms/problems. Nine patients were referred to the ED and seven for clinical management.

Comparing the patients who used the PDMC to those who did not (Table 2), the clinical characteristics were similar except for the poorer renal function, higher BI score at discharge, and less tube/catheter use in patients who did not use the PDMC. In contrast, patients with PDMC plus RFS had higher adjusted CCI score, longer hospital stay, and lower BI score than those without RFS.

**Table 2:** Clinical characteristics of the enrolled patients according to post-discharge medical counseling (PDMC) status and red-flag sign (RFS).

	Patients not using PDMC (n = 159)	Patients using PDMC (n = 65)	Patients using PDMC with RFS (n = 16)
Age, years	69.3±15.5	72.2±16.2	70.2±18.4
Sex, Male	71 (45)	21 (32)	4 (25)
Adjusted CCI score	4.9±3.1	5.6±2.6	6.8±3.2#
Primary care physician, yes	110 (69)	51 (79)	12 (75)
Marriage			
Married	115 (72)	43 (66)	10 (63)
Never married	13 (8)	8 (12)	2 (13)
Other†	26 (16)	14 (22)	4 (25)
Laboratory data, at initial admission			
Leukocyte count,/µL	10866±6420	10100±5009	9416±4864
Hemoglobin, g/dL	11.6±4.8	11.0±2.7	10.6±2.9
Creatinine, mg/dL	2.6±5.3	1.6±1.7*	1.4±1.1
Care-giver at home			
Child generation	52 (33)	30 (46)	11 (69)
Spouse	101 (64)	34 (52)	5 (31)
Other‡	6 (3)	1 (2)	0
Length of hospital stay, days	10.4±10.2	10.7±5.7	14.6±5.9#
Barthel index score at discharge	67.4±39.0	50.8±42.7*	34.4±42.3#
Artificial tube/catheter	35 (22)	24 (37)*	7 (44)
Wound needing dressing	19 (12)	7 (11)	3 (19)

Abbreviation: CCI, Charlson co-morbidity index.

Data are no. (%) or mean ± standard deviation unless otherwise indicated.

* Statistical significance (*p*<0.05) comparing patients with and without PDMC.

# Statistical significance (*p*<0.05) comparing patients with and without PDMC and RFS.

† Includes divorced patients and those who lost a spouse.

‡ Includes parents and siblings.

In the post-discharge course (Table 3), patients with PDMC plus RFS had higher rates of emergency department visits and readmissions (94% and 56%, respectively) than patients with PDMC only (48% and 29%, respectively) and those without PDMC. Unexpected death after discharge was similar in all three groups.

**Table 3:** Adverse outcomes within 30 days after discharge according to post-discharge medical counseling (PDMC) status and red-flag sign (RFS).

	Patients not using PDMC (n = 159)	Patients usingPDMC (n = 65)	Patients usingPDMC with RFS (n = 16)
Call-in day after discharge,	–	8.6±7.4	8.6±7.3
Emergency department visit	29 (18)	31 (48)*	15 (94) #
Unplanned readmission	21 (13)	19 (29)*	9 (56)#
Unexpected death	2 (1)	1 (2)	1 (6)

Data are no. (%) or mean ± standard deviation unless otherwise indicated.

* Statistical significance (*p*<0.05) comparing patients with and without PDMC.

# Statistical significance (*p*<0.05) comparing patients with and without PDMC and RFS.

**Figure 3:**
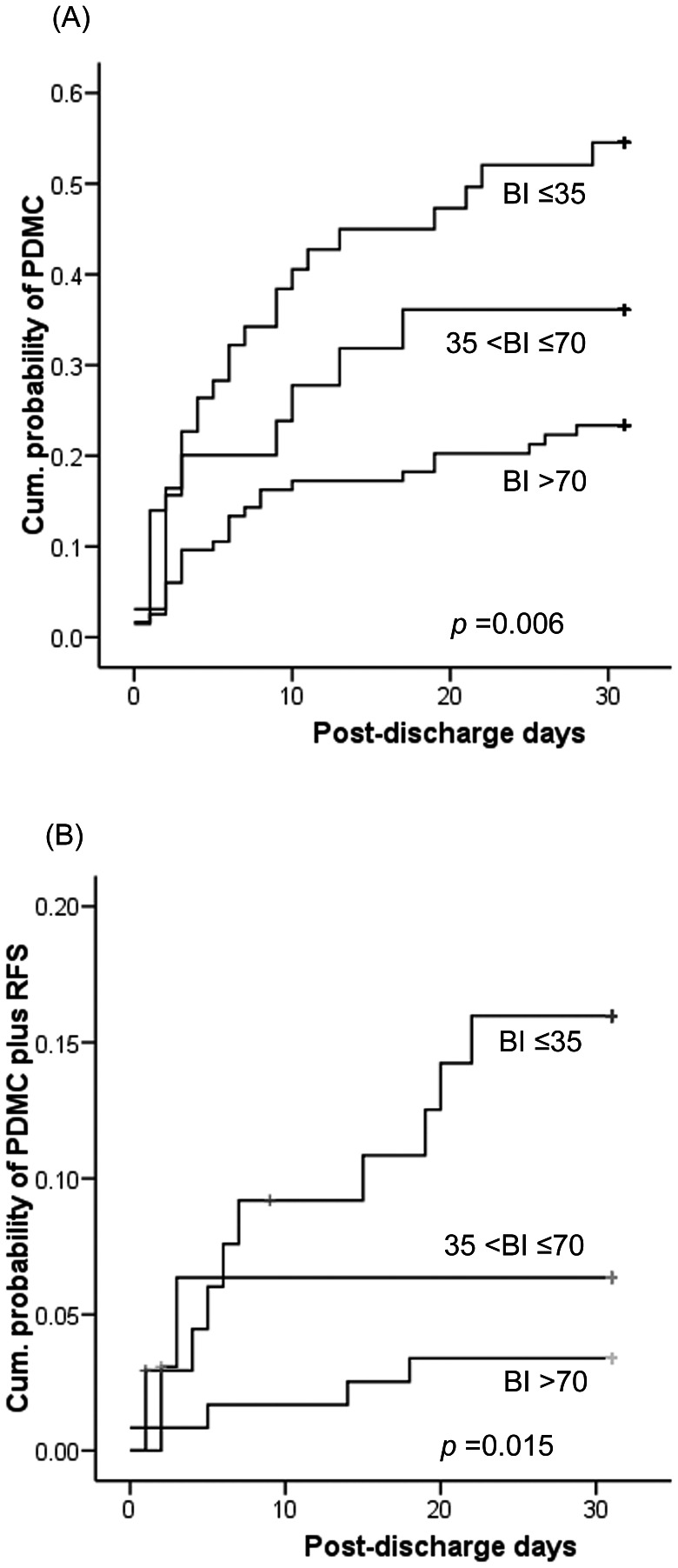
Curves plotted using the Kaplan-Meier method for (A) patients with post-discharge medical counseling (PDMC) alone or (B) with red-flag sign (RFS), according to the levels of Barthel index (BI) score.

Multivariate Cox regression was performed for PDMC within 30 days post-discharge using clinically significant factors (Table 4). The presence of underlying malignancy (Hazard ratio [HR]: 2.400; 95% confidence interval [CI]: 1.304–4.416), and BI score at discharge (HR: 0.991; 95% CI: 0.985–0.998) were independent risk factors of demand for PDMC. Sex, age, adjusted CCI score, artificial tubes/catheters, wounds requiring dressing change, and length of stay were not significantly associated with demand/use of PDMC. Regarding PDMC plus RFS, adjusted CCI score (HR: 1.207; 95% CI: 1.013–1.438), BI score at discharge (HR: 0.980; 95% CI: 0.966–0.993), and length of hospital stay (HR: 1.039; 95% CI: 1.006–1.073) were independent predictors. The associations between BI and PDMC or PDMC plus RFS were plotted and revealed statistical significance (Fig. 3).

**Table 4:** Multivariate analysis of factors associated with demand for medical counseling within 30 days post-discharge.

Characteristics		For counseling demand		For counseling with red-flag sign	
		*p* value	HR (95% C.I.)	*p* value	HR (95% C.I.)
Age, years	>65 vs. ≤65	0.739		0.073	
Sex	male vs. female	0.234		0.216	
Artificial tube/catheter	at least one vs. none	0.747		0.564	
Wound needs dressing	presence vs. absence	0.749		0.714	
Charlson score, adjusted	per unit increment	0.757		0.035	1.207 (1.013–1.438)
Barthel index score at discharge	per unit increment	0.008	0.991 (0.985–0.998)	0.003	0.980 (0.966–0.993)
Primary care physician	presence vs. absence	0.374		0.242	
Underlying malignancy	presence vs. absence	0.005	2.400 (1.304–4.416)	0.772	
Length of hospital stay	per day increment	0.505		0.020	1.039 (1.006–1.073)
Anemia*	presence vs. Absence	0.432		0.161	

Abbreviation: CI, confidence interval.

* Anemia was defined as hemoglobin <12 g/dL in males and <11 g/dL in females.

For the additional resources spent on maintaining a part-time PDMC, all of the time the staff used in the process of enrollment and medical counseling were recorded. Based on staff’s regular payment, the approximate costs spent for this service was calculated. Approximately US dollar (US$) 903.50 was used for the recruitment process and US$ 326.90 for the counseling service (exchange rate of US$ 1.00 to NT$ [New Taiwan dollar] 29.37 as of 1st October 2012) (Table 5). If the costs were divided among the 224 enrolled patients, the cost was US $5.5 per patient for PDMC use in the first month post-discharge.

**Table 5:** Related staffing costs in managing hot-line medical counseling.

Staff	Event	Time used (mean, min)	Total number	Cost/hr (US$)*	Total cost (US$)
Case manager	Recruitment and instructions	13.2	351	11.7	903.5
Attending physician	Managing counseling	3.7	54	26.0	86.6
Nurse practitioner	Managing counseling	9	30	18.8	84.6
Case manager	Managing counseling	6.6	121	11.7	155.7

All costs were at an exchange rate of US$ 1.00 to 29.37 NT$ as of 1st October 2012.

* Cost/hr was calculated using average monthly payment divided by 160 formal work-hours per month. Payment information was based on the time period from January to June 2012.

## Discussion

In the present study, the demand for PDMC is high (29%) among home care patients within 30 days post-discharge. Half of the PDMC inquiries were related to symptom management, while the rest were for care skill instructions (24%) and clinic/exam or certification arrangements (26%). With or without RFS, PDMC use was associated with higher emergency department visits and unplanned readmissions. Predictors of PDMC use were underlying malignancy and lower BI score at discharge, whereas higher CCI score, longer hospital stay, and lower BI score were predictive of RFS.

Early after discharge, a patient’s condition may dynamically and easily change. One of the reasons is poor transition of care service from the hospital setting to home care [12], [16]. Although the demand for PDMC is high, a fourth of them were requests regarding the requirements of administrative services, such as clinic/examination arrangements or certificate applications, while another one-fourth are related to the needs of care instructions such as knowledge/techniques learning. In short, care instructions and administrative services account for 50% of the counseling. For these, pre-discharge training and strengthening the way in which instructions are given can reduce the demand for PDMC and may prevent worsening of conditions. For instance, by improving post-discharge drug adherence, the occurrence of readmission can also be reduced, based on a previous report [17]. Moreover, in-patients cared for by primary care physicians may also decrease the demand for PDMC due to its lower care discontinuity [10].

On the other hand, half of the PDMCs are associated with symptom management. The proportion for symptoms counseling is similar to that of a study from the United States (14.5% vs. 13%) [18]. Among them, RFS is highly associated with post-discharge adverse events and should be considered as warning of worsening medical condition [13]. Predictors for PDMC and RFS can be used to target groups for monitoring when resources are limited. Among the predictors, activities of daily life measured by BI are associated with demands for PDMC with RFS. One possible explanation is that patients with lower BI are more dependent and have more tube/catheter and wounds, thereby requiring more care and support [19]. Poor functional status is also reported to be associated with aspiration pneumonia [20], [21] and urinary tract infection [22]. Thus, before discharge, it is important to ensure that caregivers have good care skills to improve transitional care.

Underlying malignancy instead of CCI score correlates with the use of PDMC and indicates that cancer-associated medications, symptoms, and psychological change like depression may impact on quality of care [23]. However, most PDMCs are without RFS. In contrast, similar to BI, CCI and length of hospital stay significantly correlates with PDMC plus RFS, possibly because these two factors are associated with patient complexity [11], [24].

The cost of US$ 5.50 per patient is more expensive than the US report by Rennke et al. of US$ 0.26 [18]. This may be due to the recruitment fee and high call-in rate in Taiwan. The costs may be reduced if this service is integrated into routine transitional care so that no recruitment fee will be required. Although the role of PDMC in transitional care is not investigated in the present study, it can be considered a sensor to detect this problem. A randomized controlled trial or an observational study is needed to investigate the cost-effectiveness of PDMC, especially regarding savings in resources. For those without RFS, telephone counseling may provide patients prompt answers and instructions for problems in a post-discharge care setting. However, for PDMC plus RFS, an effective strategy that can manage RFS should also be studied in the future.

There are three limitations to our study that are worth noting. First, because this study was performed in a tertiary referral center and patients have multiple co-morbidities, whether or not the results can be generalized to regional or district hospitals warrant further investigations. Second, the study uses a telephone service to investigate post-discharge adverse events and this may underestimate the occurrence. Third, the cost of equipment is not included in the present study and may underestimate PDMC cost.

In conclusion, 29% of home-care patients demand for PDMC and 7% have RFS within 30 days. Among the PDMCs, 50% are for help regarding symptom management and the other 50% are for care instructions and administrative services. The use of PDMC is associated with emergency department visits and unplanned readmissions. Pre-discharge care instructions should be emphasized in patients with cancer and low BI. Patients with lower BI, higher CCI, and longer hospital stay are a risk population for RFS after discharge and require other types of support like transitional care.
